# The influence of rare birds on observer effort and subsequent rarity discovery in the American birdwatching community

**DOI:** 10.7717/peerj.10713

**Published:** 2021-01-21

**Authors:** Jesse A. Laney, Tyler A. Hallman, Jenna R. Curtis, W. Douglas Robinson

**Affiliations:** 1 Department of Integrative Biology, Oregon State University, Corvallis, OR, United States of America; 2 Department of Fisheries and Wildlife, Oregon State University, Corvallis, OR, United States of America; 3 Cornell Lab of Ornithology, Cornell University, Ithaca, NY, United States of America

**Keywords:** eBird, Patagonia Picnic Table Effect, Birding, Citizen science, Birdwatching, Rare birds, Birder behavior, Crowd-sourced data, Twitching, Twitchers

## Abstract

Birdwatching is a rapidly growing pastime, increasingly involving the pursuit of rare birds as birders build lists of species they encounter. We expected reports of rare bird discoveries to quickly draw birders to locations, and that the increased attention at those locations would decay over time. We hypothesized that magnitude of draw and rates of decay would vary depending on characteristics of the species and the geographic locations where rarities were discovered. Discoveries of additional rarities might affect both the draw and decay, so we also quantified empirical evidence for the *Patagonia Picnic Table Effect* (PPTE), a social feedback loop where rarity discoveries are presumed to lead to additional rarity discoveries because of the elevated levels of birder activity once an initial rarity is reported. Although commonly invoked, supporting evidence for the PPTE hypothesis is anecdotal. We used 10 years of eBird data (2008–2017) in the United States to (1) understand birding activity when rarities were reported and the factors associated with draw and decay, and (2) assess the frequency at which initial rarity discoveries lead to reports of additional rarities. Across 273 rarity events, birder effort, as indexed by numbers of eBird checklists, increased above the pre-event baseline level, with the magnitude of draw varying geographically. We found no indication that draw was influenced by species identity or rarity-level, but latitude and distance to small airport proved important in drawing additional eBirders to rare bird discoveries. Mean draw of rarities and mean number of checklists from the same locations prior to each rarity discovery grew through the ten years, suggesting an increased influence of eBird on birder behavior in general. Decay rates in birder effort were more gradual in rare bird events with longer durations. Effort declined below baseline-levels after rarities went undetected, suggesting, “location-fatigue” following rarity events. Results did not support the PPTE hypothesis. Controlling for site-specific circumstances, birders had no better chance of finding additional rarities during events than at times outside events. Our results emphasize that eBird checklist quantity at rarity events follows a predictable but variable pattern of draw and decay influenced by location and time since rarity discovery; that birders have statistically similar chances of finding rarities during normal “baseline” birding activities as they do when known rarities are present; and that eBird represents a largely untapped resource for studying factors that influence levels of birding activity.

## Introduction

Birdwatchers, otherwise known as birders, are one of the fastest growing public groups with special interests in biological diversity (Cordell & Herbert, 2002; Wood et al., 2011). In the United States alone, birders contribute billions of dollars to the economy each year, largely from expenditures for supplies and travel; and they are influential voices in their communities, affecting environmental policies (McFarlane & Boxall, 1996; Carver, 2009; Carver, 2011; USFWS, 2016). Therefore, understanding their activities and motivations can improve conservation efforts, assessment of their scientific contributions, and identify methods to motivate engagement with ornithological research (Tarrant, Bright & Cordell, 1997; Ma et al., 2013; Steven et al., 2017; Bennett et al., 2017).

Factors motivating birders vary according to level of experience (Kim, Scott & Crompton, 1997; Cole & Scott, 1999; Hvenegaard, 2002; Scott & Thigpen, 2003; Eubanks, Stoll & Ditton, 2004). Most birders engage casually with birds, focusing on enhancing their own property to attract more birds (McFarlane & Boxall, 1996; Carver, 2009; Carver, 2011), watching or photographing birds for pleasure during outdoor activities (McFarlane, 1994; McFarlane & Boxall, 1996), or joining organized activities such as Christmas Bird Counts (McFarlane & Boxall, 1996; Eubanks, Stoll & Ditton, 2004). A growing proportion of the birding community, however, is more actively engaged with seeking out species of interest and keeping records of their birding activities (Sheard, 1999). Such birders build lists of species encountered and organize those lists to track numbers of species encountered during their own lifetime, a given year, or within particular geographic areas of interest (Wood et al., 2011). For example, vagrant birds, or *rarities*, represent opportunities for birders to observe species that have wandered outside their typical geographic range (Brock et al., 2020). Birders around the globe invest significant time and effort to observe rarities and add them to their personal lists (Booth et al., 2011; Straka & Turner, 2013; Callaghan et al., 2018; Callaghan et al., 2019; Brock et al., 2020). It is this group of birders who “chase” rarities (also known as “twitchers” in some parts of the world) (Connell, 2009; Brock et al., 2020) that is the focus of our study.

### Rarities and birder behavior: draw and decay

**Figure 1:**
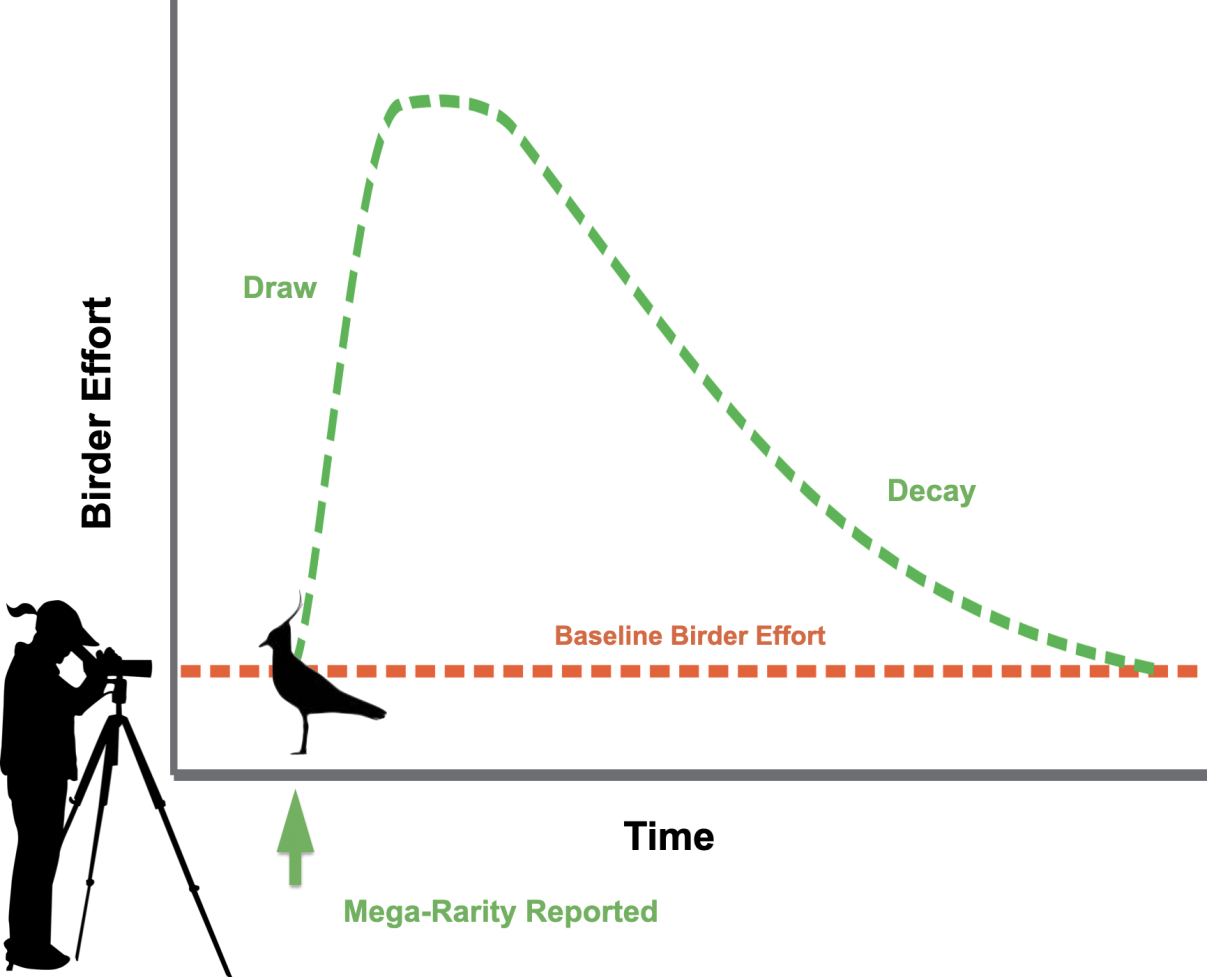
Hypothesized behavioral dynamics of birders when rare species are discovered. Dashed green line represents birding effort when rarities have been discovered, including the *draw* following initial report of rarity and the *decay* in effort over time. Dashed orange line represents the baseline birding effort at a given location. Northern Lapwing silhouette clipart ©Adobe Stock. Birder silhouette clipart source: https://openclipart.org/detail/222259/lady-spotting-scope.

We sought to understand the behavioral dynamics of birders who contribute observations to the online database, eBird (Sullivan et al., 2009; Sullivan et al., 2014), when rare species are detected. In eBird, birders submit checklists created for each outing, noting the location, date, start time, duration of effort spent at the site looking for birds, a list of all species detected, and, ideally, a count of the number of individuals of each species. Checklists, therefore, provide a measure of the amount of effort birders have spent at a site looking for birds on a given date. We hypothesized that some rare bird discoveries attract more attention than others due to their attractiveness to birders and result in increased birder effort. We call this level of attraction the *draw* (Fig. 1). The draw is therefore reflected as an increase in the number of eBird checklists submitted in a given location where a rarity is first discovered above the average number of checklists submitted in the same location before the discovery of the rarity. Typically, within minutes of an initial discovery, reports of rare birds are shared via regional list-serves, social media, texting services, and rare-bird “needs” e-mail alerts from eBird (Straka & Turner, 2013). Birders generally know the chance of seeing a rare species decreases quickly as time after an initial report elapses because most birds are quite mobile, and many can be difficult to re-locate. Thus, we should expect birder behavior to respond dynamically as time passes following reports of rarities. We hypothesized that increased observer-effort resulting from the draw should eventually return to the baseline-level of effort prior to the rarity report due to either the disappearance of the rare bird or reduction of interest over time. We call the subsequent decline in birder effort following a peak the *decay* (Fig. 1), which eventually returns to the checklist submission rate prior to rarity detection. The occurrence of draw and decay are logical observations of birder activity patterns. The factors influencing the quantitative values of draw and decay, however, are as yet unstudied.

The magnitude of draw and rate of decay could be influenced by several factors including the relative rarity of the species. For example, first country records, otherwise known to birders as ‘mega-rarities,’ are particularly valued by birders who spend considerable time, money, and effort to observe them (Brock et al., 2020). Thus, mega-rarities might attract far more birder activity than a first state or province record, or a first county or parish record, and such discoveries may sustain increased effort for longer durations. Species-specific factors, such as body size or taxonomic classification, might also contribute to their attractiveness to birders (Schuetz & Johnston, 2019). Spatial factors such as relative ease of access and proximity to a large city could also influence the number of birders able or willing to seek a rarity. We predicted that the rate at which eBird checklist numbers decayed would be influenced by similar factors affecting the draw. Specifically, we predicted greater draw for rarities considered as rarer, and that those rarities would sustain more birder attention for longer (lower decay rate) than lesser rarities. We predicted greater draw for rarities discovered at sites with easier access and nearer to large population centers than rarities at more rural locations, and that rare species detected at sites closer to population centers with easier access would exhibit lower decay rates.

### Does one rarity discovery lead to another?

A possible influence on the decay of birder activity could include subsequent discoveries of additional rarities after the initial rarity draws birders to a location. In North America, the phenomenon of finding new rarities while chasing a previously reported rarity is known among birders as the *Patagonia Picnic Table Effect* (PPTE). Possibly coined by well-known birder, P. William Smith, after the discovery of one first U.S. record at a roadside rest stop near the town of Patagonia in southern Arizona led to the subsequent discovery of yet another first country record, the phrase has since become a neologism for the tendency of one rare bird observation to lead to another at any given geographical location (Kaufman, 2006; Kaufman, 2020). Use of the PPTE locution to describe the presumed phenomenon of one rarity leading to the discovery of at least one additional rarity at the same geographic location has become widespread in birder vernacular in North America and is commonly evoked whenever any site hosting a rare bird attracts many birders chasing it. Similar phenomena occur elsewhere, though they may go by different names in other birding cultures. Nevertheless, supporting evidence for the supposed effect of rare bird discoveries leading to additional rarity discoveries has thus far been anecdotal.

We sought to understand the frequency with which the PPTE phenomenon, rarities leading to discovery of additional rarities, actually happens. Though the PPTE is often referenced in popular birding publications and ornithological field-note reports, it is rarely mentioned in peer-reviewed literature (but see Brush, 2014; Dinets, 2016). Because appearance of additional rarities could influence our characterization of draw and decay, we evaluated the occurrence and potential effect of the PPTE. To our knowledge ours is the first study to quantitatively investigate the phenomenon. To do so, we formally characterized it as a testable hypothesis, wherein rare bird discoveries lead to discovery of at least one additional rarity at the same geographic location because of the increased attention and effort of birdwatchers. To test this hypothesis, we used the number of eBird checklists submitted in given geographic locations and the species records contained therein to understand the frequency with which the discovery of one rare species led to subsequent discovery of another rare species. Evaluation of this hypothesis requires control of geographical circumstances because rarities are not randomly distributed. Instead, within the United States, records of the rarest species tend to be aggregated along the US-Mexico border and in Florida. Those locations also have overall higher numbers of rare species in their bird communities because of their geographical locations. Because context is critical, an effective test of the hypothesis requires controlling for these factors in each case. Comparing rates of discovery of additional rarities with the ambient, or baseline, level of rarity discovery rates for each specific site is required to test the PPTE hypothesis. Paradoxically, a continent-wide rarity discovery rate as a point of comparison would provide an ineffective evaluation because most places do not attract extremely rare birds, so the ambient rarity discovery rate would be biased unreasonably low. By controlling for geographic location, we were able to test whether the PPTE hypothesis is supported by eBird observations or if it is simply a myth created after one particularly noteworthy historical event. Collectively, our evaluations of the PPTE and the factors affecting draw and decay of birding activities associated with rare birds are the first to quantitatively characterize these common birder activities.

## Methods

### Defining mega-rarities

We focused our investigations on discoveries of the rarest of birds in the United States, otherwise known as mega-rarities. The American Birding Association (ABA) places bird species into five abundance categories (5 being rarest, 1 being most common). We defined mega-rarities as ABA code-5 and code-4 rare species. Species in code-5 consist of species that have been recorded five or fewer times in the ABA checklist-area, or fewer than three records in the past 30 years. Code-4 species are those that are not recorded annually in the ABA’s checklist-area but have six or more total records including three or more recorded in the past 30 years. The ABA checklist-area encompasses the 49 continental U.S., Hawaii, Canada, the French islands of St. Pierre et Miquelon, and adjacent waters to a distance of 200 miles from land or half the distance to a neighboring country, whichever is less (American Birding Association, 2017).

### Defining mega-rarity events with eBird data

We generated a comprehensive dataset of eBird checklists containing discoveries of mega-rarities within the continental United States during a 10-year time period: 2008–2017 (eBird, 2017a; eBird, 2017b). Among other methods, eBird incentivizes participation by keeping track of birders’ life lists and sending out rare bird alerts. As such it is the ideal source of data for our study. First, we used eBird’s ‘species map’ tool to query all occurrences of mega-rarities since 2008. We used this year as a cutoff because of a lower number of active eBird members prior to 2008. We limited our search to terrestrial locations within the continental U.S. and near-shore islands (i.e., no sea-watch observations or checklists from pelagic-birding trips were included). By visually assessing checklist clusters on maps generated by our searches, we sorted and classified mega-rarity observations into distinct *events*. Each event was characterized by the birding activity associated with its focal species. The dates of the first and last checklists that contained an observation of the event’s corresponding focal species defined the temporal bound of each rarity occurrence event. In very rare cases where multiple occurrences of the same rare species overlapped in time, we also used geographic clustering of checklists to distinguish each event.

To enable investigation of draw and decay, we downloaded relevant county-level eBird data for a period beginning two-weeks prior to the date of the first checklist that contained the event’s corresponding focal rarity, and ending six-weeks following the date of the last checklist that contained the focal rarity (a total of 56 days surrounding each event). We categorized these data into three distinct phases: before event, during event, and after event. These before and after event-start phases were analogous to experimental control and intervention in our analyses of draw vs. decay, and estimation of rarity detection rates. Estimates of baseline birding activity before and after rarity events must be site-specific, linked directly to each event, because each location has different bird communities and different circumstances that influence probabilities of rarity occurrence.

To ensure that the signal of the effects of a rare bird event on birder effort could be identified, we further constrained eBird checklists to locations immediately surrounding events. We used package *ArcPy* in Python (ArcGIS, ESRI Corporation, Redlands, CA) to clip eBird data to geographic buffers around the coordinates of the first checklist in each data file that contained the mega-rarity. We used a buffer of 9 km, which is approximately two standard errors away from the mean distance traveled by observers who first submitted checklists of a rarity and used traveling protocol. Occasionally, mega-rarity events spanned geographical areas that crossed county or state lines. This posed a problem because the eBird checklist data used in our dataset was constrained to the county level. Thus, we constrained events to those that did not span multiple counties or states. To account for shared checklists among eBird users, we removed duplicate checklists within events.

Certain mega-rare species were associated with a higher number of events than others (e.g., Barnacle Goose (*Branta leucopsis*): *n* = 109 vs. Pink-footed Goose (*Anser brachyrhynchus*): *n* = 3; see Table S1 for complete list). Though code-4, the relatively frequent events of some species compared to others could dilute the effects of mega-rarities. To avoid a single species driving patterns and any dilution of an effect, we limited our sample size to include a maximum of 10 randomly selected events per mega-rare species.

To ensure accurate interpretation of each event, we removed events from the dataset when two or more discoveries of the same mega-rarity species were present in the same county during overlapping time periods and disentangling birding activity associated with each event was problematic (*n* = 41 events). We also removed events where the 9 km radius buffer appeared too small for the event because checklist detections were too greatly distributed (*n* = 4 events), or in cases where events had strange effort patterns (suggestive of banding stations; *n* = 3 events). Using these criteria, we generated a dataset of 273 distinct mega-rarity events representing 81 distinct focal species.

### Modeling temporal patterns of checklist submission rates

To assess the relationships between number of checklists submitted per day during rare bird events and the comparative influence of temporal covariates (see below), we used generalized linear mixed-effects models. Event ID was treated as a random effect to account for the lack of independence in the number of checklists submitted each day within each event. Due to the high number of zeros in our data, negative binomial provided a better model fit than Poisson and was used in these analyses. Before running models, we checked for multicollinearity and removed covariates that were correlated by more than *r*^2^ = 0.5. We included models with variables independently and added complexity by combining covariates that outperformed the null model. We used Bayesian information criterion (BIC) to select the top performing mixed-effects model.

Temporal covariates in our models included event year, categorical day of the week, categorical weekend or weekday, Julian date, event duration, event day (i.e., the day number since event start), and categorical phase. For phase, we considered models that included three phases—before (14 day phase before to first checklist detection of the focal rarity); during (the duration phase of the event itself); and after (the 42 day phase following the last checklist detection of the focal species) and models that included only two phases—the event phase and a combined 56-day before and after phase surrounding the event. For event day, we also included quadratic and cubic terms to allow for nonlinear relationships. Similarly, for Julian date we included a quadratic term.

### Modeling event draw, event decay rate, and checklists before events

To estimate the draw of each event, we calculated the mean number of checklists submitted per day during the 14-day before phase and then subtracted this before phase checklist submission rate from the maximum number of single-day checklists submitted within the first seven days of an event. We used the maximum value within the first week of an event to help control for noise associated with higher numbers of checklists submitted on different days of the week (i.e., weekends vs. weekdays).

We used linear model sets to describe the influence of temporal, spatial, and species-specific covariates on our estimations of draw across all events in the dataset. We used a base-10 log transformation of draw to normalize the response variable. Covariates included Julian date, quadratic Julian date, latitude, longitude, population of the nearest city, distance to the nearest city, distance to the nearest urban area, distance to the nearest small, medium, and large airport, county population during the event (United States Census Bureau, 2020), and the dominant land cover classification in a 1 km radius of the original event location. NLCD land cover classifications, airport locations and enplanement data from the federal aviation administration, and shapefiles of city population and urban area from the Natural Earth Dataset (http://www.naturalearthdata.com/) were used to calculate spatial covariates. We classified airports into small, medium, and large based on enplanement values of less than or equal to 50,000, 250,000, and 1,000,000. Species-specific covariates consisted of the event’s focal species itself, taxonomic order, taxonomic family, body size measured as average species length in cm (Billerman et al., 2020), and rarity-level as defined by ABA code (American Birding Association, 2017). As with the mixed effects models, highly correlated variables were removed prior to running models. We used BIC to determine the top-performing models for event draw.

To estimate the decay in birder effort following the initial draw of each event, we calculated the slope of an exponential decay model from the event start date until 14 days following the event end date (i.e., the last day the rare bird was detected). Choosing a 14-day period following each event helped to better-fit exponential decay models by providing more of an asymptote via more overall data. Seven events had excessively high exponential slopes that skewed the data and were treated as outliers, and an additional 14 events could not be fit using exponential decay models (see Fig. S1 for examples). After removing these 21 events, we used a base-10 log transformation to normalize data in the model set of exponential decay. We then used linear model sets to investigate the same temporal, spatial, and species-specific covariates on the rate of decay as were used for modeling draw. We used BIC to determine the top-performing models for rate of decay. While decay rate is an exponential function, we also calculated decay using linear models and a base-10 log transformation for normalization. Doing so allowed us to avoid having to remove 21 events. The linear model set yielded similar results (see Table S1).

We also investigated the relative influence of the same temporal, spatial, and species-specific covariates on checklist submissions before each rarity event. We summed the checklists to get a count of number of checklists submitted in the two weeks prior to the rare bird discovery for each event. We used Poisson GLMs and conducted BIC model selection to determine the top-performing models for checklist submissions before events. As with all above model sets, correlated variables were removed prior to analysis using a 0.5 level as a cutoff (see Table S1).

### Comparing rarity discovery rates

In order to investigate our hypothesis that increased birder effort during events led to the discovery of additional rarities (i.e., the PPTE), we estimated both daily and per checklist rarity discovery rates for geographic locations during events, as well as the baseline rates at those same locations before and after events. The baseline included a total of 42 days surrounding each event that were composed of a two-week period directly before the first checklist containing the focal rarity, and a four-week period that began 14 days after the last checklist that contained the focal rarity. We did not include the two weeks directly following events in our baseline because we assumed when the last checklist including the focal rarity was made, birders could not yet know that this was indeed the last checklist, and hence we assumed they should not immediately behave differently. Similarly, we included the first week following the last occurrence of the focal rarity in our estimation of during-event effort because of a strong influence of weekends on checklist submissions; therefore, we assumed the week immediately following the last rarity observation was likely still influenced by that event.

To calculate the baseline daily rarity discovery rate for each event, we summed the number of rare species observed during the baseline period surrounding the event and then dividing this sum by the total days used as the baseline. Similarly, we calculated the event daily rarity discovery rate by dividing the number of additional rare species found during the event by the total duration of the event (i.e., total number of days from first to last detection of the focal rarity + 7 days immediately after last detection). To calculate the baseline per checklist rarity discovery rate for each event, we divided the number of rare species discoveries in the baseline period surrounding each event by the total number of checklists in that period. For the event per checklist rarity discovery rate, we divided the number of non-focal rare species by the number of checklists submitted within an event and the first 7 days after last detection of the focal rarity. We used paired *t*-tests, to compare baseline and event (1) daily rarity discovery rates, and (2) per checklist rarity discovery rates.

ABA code-4 and code-5 species are rare everywhere in our study area, so should stimulate rarity-chasing behavior among birders. In contrast, code-3 includes many species whose level of rarity is more contextual and may not elicit the same behavior. These contexts vary geographically, annually and even seasonally, which is why we defined our events based on code-4 and code-5 species. However, in many instances the discovery of a code-3 species during a chase for a code-4 or code-5 species would be considered by most of the birding community to be a discovery of a new rarity, again, with many exceptions. Therefore, we included code-3 species in our calculations if such species were found subsequent to discovery of a code-4 or code-5 species.

## Results

Our analysis of 273 separate events initiated by the discovery of 81 focal mega-rarity species contained 119,623 eBird checklists. Of those checklists, we found 12,469 that documented detections of mega-rarity species. Event length ranged from 1 to 416 days with a median duration of 8 days and a mean duration of 28.6 days (standard deviation = 49.3). The events occurred across 31 states in the U.S., yet over half were near the U.S.-Mexico border and the Gulf Coast, with 18.3% having occurred in southern Arizona, 13.9% in the Rio Grande Valley and coastal Texas, and 18.7% in Florida. The events were evenly distributed across calendar years and occurred in a wide range of landscapes and areas of varying human-population densities and proximities to urban centers. The 81 focal mega-rarity species belonged to 40 separate taxonomic families classified within 13 orders and ranged in body length between 8–131 cm (see Table S1 for all variables).

### The shape of rare bird events over time

**Figure 2:**
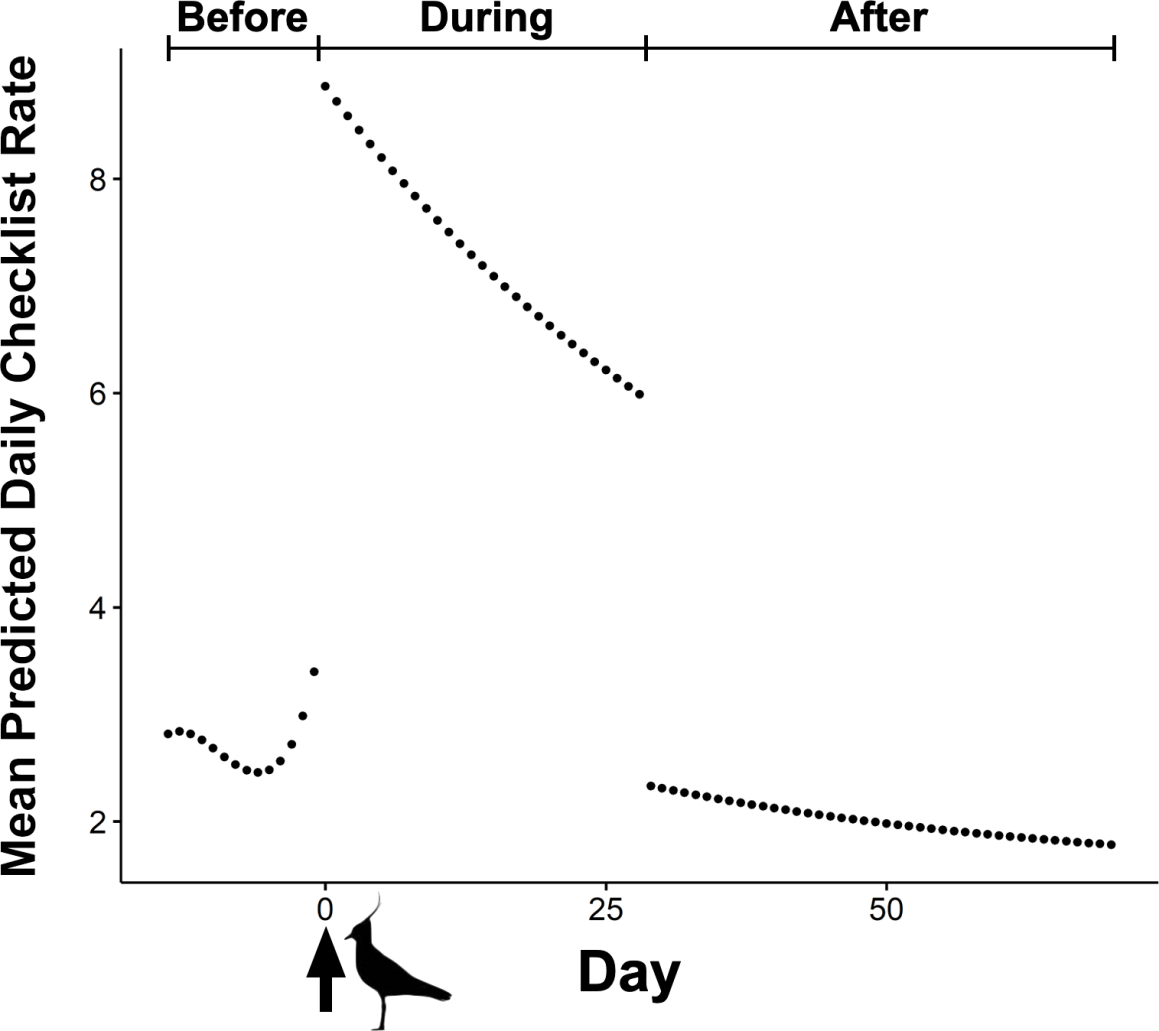
Mean predicted daily checklist submission rate before and after the first eBird report of corresponding rare species during 273 ABA code-4 and -5 mega-rarity bird events. Arrow at day zero represents the first eBird report of focal rarity. Before, During, and After indicate the three event phases included in the top performing mixed-effects model (BIC weight = 0.99). Northern Lapwing silhouette clipart ©Adobe Stock.

The BIC top model in our mixed effects model set held over 99 percent of model weight and was over 20 BIC from the next competitive model (Table 1). The top model included an interaction between the categorical effect of phase during an event (i.e., before, during, and after) and the cubic effect of event’s day number, as well as additive effects of day of the week, and event year (Table 1). The predicted shape of an event with a mean duration of 29 days shows the three distinct phases (Fig. 2). Following the initial draw, mean daily checklist rate steadily decayed through the duration of the events until 30 days after initial detection of rarities, where we then observed a large drop in mean daily checklist rates—below that of before-phase rates—followed by a shallower, but continued decay in checklist rates (Fig. 2). The influence of the day of week varied by day where the highest number of checklists were predicted on Saturday, followed by Sunday and Friday (Table 2).

**Table 1:** Top ten linear mixed models of number of checklists submitted during rare bird events ranked by Bayesian information criterion (BIC). Models are based on 273 ABA code-4 and -5 rare bird events with the unique ID assigned for each event treated as the random effect in a negative binomial distribution. For the complete table of models with rankings (*n* = 51) and relative variable importance of top model, *n* = 18, see Supplementary Information).

Ranking	Fixed effects	Log-likelihood	BIC	Δ_*i*_	*w*_*i*_
**1**	Event Phase*(Day Number + Day Number^2^+ Day Number^3^) + Day of Week + Year	**−47,075.968**	**94,362.935**	**0**	**0.999**
2	Event Phase*(Day Number + Day Number^2^+ Day Number^3^) + Weekend + Year	−47,112.327	94,385.415	22.480	<0.001
3	Event Phase + Day Number + Day Number^2^+ Day Number^3^+ Day of Week + Year	−47,118.471	94,387.655	24.720	<0.001
4	Event Phase*(Day Number + Day Number^2^+ Day Number^3^) + Day of Week	−47,101.264	94,403.481	40.545	<0.001
5	Event Phase + Day Number + Day Number^2^+ Day Number^3^+ Weekend + Year	−47,155.706	94,411.888	48.953	<0.001
6	Event Phase + Day Number + Day Number^2^+ Day of Week + Year	−47,115.846	94,412.549	49.614	<0.001
7	Event Phase*(Day Number + Day Number^2^+ Day Number^3^) + Weekend	−47,137.551	94,425.815	62.880	<0.001
8	Event Phase + Day Number + Day Number^2^+ Day Number^3^+ Day of Week	−47,143.557	94,427.781	64.845	<0.001
9	Event Phase*(Day Number + Day Number^2^) + Weekend + Year	−47,152.093	94,434.805	71.870	<0.001
10	Event Phase*(Day Number + Day Number^2^+ Day Number^3^)* Weekend * Year	−47,180.715	94,451.859	88.924	<0.001

**Notes.**

Δ_*i*_ is the delta BIC (difference between the BIC for a given model *i* and the best fitting model) and *w*_*i*_ is the model selection probability (probability that model *i* is the best model given the *a priori* model set).

**Table 2:** Coefficients of the top ranked linear mixed model of number of checklists submitted during rare bird events.

Coefficients	Estimate	Std. Error	*t* value	Pr (>|*t*|)
(Intercept)	1.3	0.0708E	18.393	<0.001
After Phase	−0.901	0.0237	−37.955	<0.001
Before Phase	925	0.0519	17,837.462	<0.001
Day Number	−41.2	1.95	−21.105	<0.001
Day Number^2^	35.2	1.65	21.258	<0.001
Day Number^3^	−11.7	1.68	−6.974	<0.001
Day of Week: Monday	−0.141	0.0285	−4.943	<0.001
Day of Week: Saturday	0.301	0.0275	10.954	<0.001
Day of Week: Sunday	0.194	0.0278	6.975	<0.001
Day of Week: Thursday	−0.101	0.0284	−3.535	<0.001
Day of Week: Tuesday	−0.201	0.0287	−6.987	<0.001
Day of Week: Wednesday	−0.158	0.0286	−5.532	<0.001
Year	0.498	0.0670	7.44	<0.001
After Phase*Day Number	17.1	2.24	7.651	<0.001
Before Phase*Day Number	531,000	3	177,045.301	<0.001
After Phase*Day Number^2^	−9.99	2.32	−4.298	<0.001
Before Phase*Day Number^2^	483,000	4.42	109,070.052	<0.001
After Phase*Day Number^3^	−4.34	2.14	−2.033	0.042
Before Phase*Day Number^3^	118,000	3.72	31,739.106	<0.001

### Predictors of event draw

The BIC top model for draw held over 74 percent of model weight and was over two BIC from the next competitive model (Table 3). The top model had an overall positive effect of year, negative effect of distance to nearest small airport, and a positive effect of latitude (Table 4).

**Table 3:** Linear models of rare bird events’ *draw* (maximum daily number of checklist submissions in first 7 days), exponential *decay* models (the slope of each event’s regression), and linear models of before event checklists (total checklists) ranked by Bayesian information criterion (BIC). Linear models of before event checklists and draw are based on 273 ABA code-4 and -5 rare bird events. Exponential decay models are based on 251 ABA code-4 and -5 rare bird events. Only models whose *w*_*i*_ were above 0.01 are displayed (for complete table see Supplementary Information).

Ranking	Coefficients	Log-likelihood	BIC	Δ_*i*_	*w*_*i*_
Event Draw					
**1**	**Year + Distance to Nearest Small Airport + Latitude**	**−357.539**	**743.126**	**0**	**0.745**
2	Year + Distance to Nearest Small Airport + Population of Nearest City	−356.064	745.785	2.659	0.197
3	Year + Distance to Nearest Small Airport	−362.959	748.357	5.231	0.054
4	Year + Population of Nearest City	−366.511	755.459	12.333	0.002
5	Year	−369.491	755.809	12.683	0.001
Event Decay					
**1**	**Event Duration**	**−332.067**	**680.723**	**0**	**0.653**
2	Event Duration + Distance to Nearest Airport	−330.497	683.112	2.389	0.198
3	Event Duration + Latitude	−331.209	684.536	3.813	0.097
4	Event Duration + Julian Date + Julian Date^2^	−329.262	686.171	5.448	0.043
5	Event Duration + Julian Date + Julian Date^2^+ Distance to Nearest Airport	−327.974	689.124	8.401	0.009
Before Event Checklists					
**1**	**NLCD + Year + Julian Date + Julian Date^2^+ Distance to Nearest Small Airport + Population of Nearest City**	**−6,900.317**	**13,924.042**	**0**	**0.998**
2	NLCD + Year + Julian Date + Julian Date^2^+ Distance to Nearest Small Airport	−6,909.516	13,936.831	12.790	0.002

**Notes.**

Δ_*i*_ is the delta BIC (difference between the BIC for a given model *i* and the best fitting model) and *w*_*i*_ is the model selection probability (probability that model *i* is the best model given the *a priori* model set).

**Table 4:** Coefficients of top models of event *draw* (initial daily rate of checklist submission), event *decay rate* (the exponential decay of each event), and number of checklists submitted before events.

**Coefficients**	**Estimate**	**Std. Error**	***t* value**	**Pr (>|*t*|)**
Event Draw (BIC = 743.126, *w*_*i*_ = 0.745)				
(Intercept)	<−0.001	0.055	0	1
Year	0.345	0.055	6.29	<0.001
Distance to Nearest Small Airport	−0.250	0.057	−4.419	<0.001
Latitude	0.187	0.057	3.301	0.001
Event Decay (BIC = 680.723, *w*_*i*_ = 0.653)				
(Intercept)	<−0.001	0.057	0	1
Event Duration	0.424	0.057	7.408	<0.001
Before Event (BIC = 13924.042, *w*_*i*_ = 0.998)				
(Intercept)	3.887	0.076	51.035	<0.001
NLCD Cultivated Crops	−0.356	0.084	−4.246	<0.001
NLCD Deciduous Forest	−1.55	0.152	−10.197	<0.001
NLCD Developed, High Intensity	−0.208	0.108	−1.921	0.055
NLCD Developed, Low Intensity	−0.171	0.085	−2.011	0.044
NLCD Developed, Med. Intensity	−0.092	0.088	−1.049	0.294
NLCD Developed, Open Space	0.100	0.092	1.094	0.274
NLCD Dwarf Scrub	1.688	0.186	9.098	<0.001
NLCD Emergent Herb. Wetlands	−0.560	0.100	−5.587	<0.001
NLCD Evergreen Forest	0.499	0.080	6.272	<0.001
NLCD Grassland/Herbaceous	−0.404	0.102	−3.951	<0.001
NLCD Mixed Forest	−1.139	0.160	−7.127	<0.001
NLCD Open Water	−0.170	0.078	−2.193	0.028
NLCD Pasture/Hay	−0.644	0.102	−6.401	<0.001
NLCD Sedge/Herbaceous	−2.384	0.583	−4.086	<0.001
NLCD Shrub/Scrub	−0.956	0.084	−11.372	<0.001
NLCD Woody Wetlands	−0.410	0.083	−4.939	<0.001
Year	0.417	0.011	38.18	<0.001
Julian Date	−0.889	0.183	−4.852	<0.001
Julian Date^2^	−3.107	0.176	−17.679	<0.001
Distance to Nearest Small Airport	−0.223	0.017	−13.477	<0.001
Population of Nearest City	0.038	0.008	4.426	<0.001

### Predictors of event decay

The BIC top model for exponential decay held over 65 percent of model weight and was over 2 BIC from the next competitive model (Table 3). This top model included a positive effect of event duration on the slope coefficients from models of exponential decay. This indicated that decay rate was moderated by longer events, where rate of event decay became more gradual as event durations increased (Table 4). Here, we only present results from our exponential models of decay as we felt they were a better fit of the data, however linear model set results of event decay yielded similar results (see Supplemental Information).

### Predictors of before-event checklists

The top BIC model for before-event checklist submission rate held over 99 percent of model weight and was over 12 BIC from the next competitive model (Table 3). This model included effects of national land cover classification, a negative effect of quadratic Julian date, a negative effect of distance to nearest small airport, a positive effect of year, and a positive effect of population of nearby city on the mean total number of checklists before the beginning of rare bird events across all locations (Table 4).

### Effects of rare bird events on additional rarity detection rate

**Figure 3:**
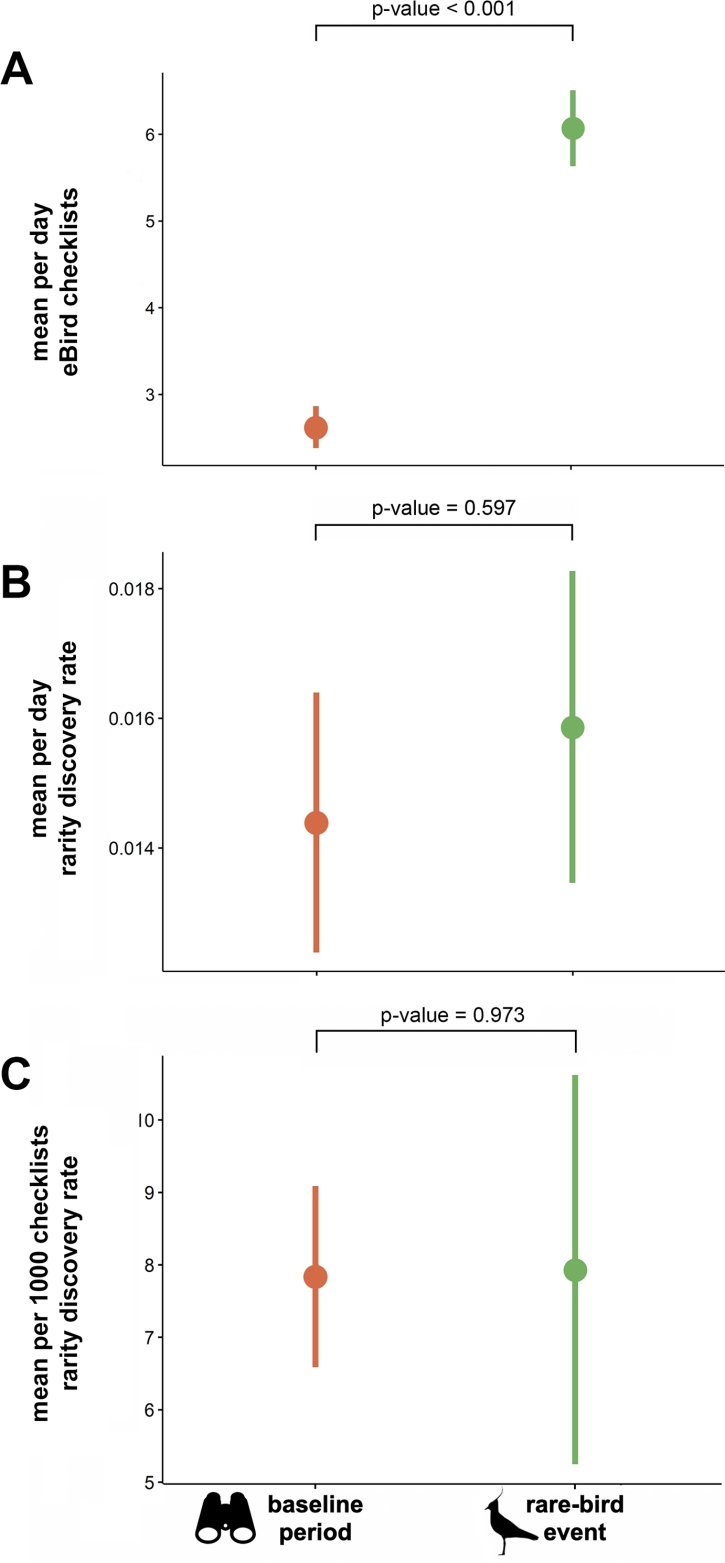
Mean eBird checklists and rarity discovery rates in baseline period vs. rare-bird events. (A) Mean number of eBird checklists submitted per day, (B) mean estimated rate of rare species discoveries per day in eBird checklists, and (C) mean estimated rate of rare species discoveries per 1000 eBird checklists submitted both during events and in the baseline period (before and after events). *P*-values indicates results of paired *t*-tests with significance level of *α* = 0.05. Northern Lapwing silhouette clipart ©Adobe Stock. Binocular clipart source: https://www.flaticon.com/free-icon/binoculars_891887#.

The mean total number of checklists submitted per day was significantly higher during rarity events than during the baseline periods (*t* =  − 12.349, *df* = 272, *p*-value <0.001; Fig. 3A). We found no significant difference in daily rarity discovery rate between eBird checklists submitted during mega-rarity events and those submitted during the baseline period surrounding events (*t* =  − 0.530, *df* = 272, *p*-value = 0.597; Fig. 3B). We also found no significant difference in rarity detection rate per 1000 checklists submitted during mega-rarity events (8 per 1,000) than those submitted during the baseline period surrounding events (*t* =  − 0.034, *df* = 272, *p*-value = 0.973; Fig. 3C).

## Discussion

### eBird and the draw and decay of rare bird events

Our top performing mixed-effects model was consistent with our hypothesis (Fig. 1) regarding the change in birder effort following discoveries of rare birds. We found a greater mean number of eBird checklists immediately following reports of rarities at the beginning of events (i.e., the draw), followed by declining numbers of checklists until the end of events (i.e., the decay) (Fig. 2). The curve shape that emerged when we modeled these data supports our expectation that large numbers of birders converge upon locations where rarities have recently been reported, and over time this unusually high level of birder-effort and attention decreases as more observers successfully chase and observe the rare bird, leading to depletion of the pool of observers still investing birding effort at each site. We found that day of week appears to be an influential factor when describing birder behaviors during rarity events. Though previous work (Courter et al., 2013) has indicated that weekend bias in crowd-sourced bird data reporting has decreased over time in North America, our results show that weekends were particularly important for explaining eBirder dynamics over the 10 years covered in our study as the highest number of checklists during rarity events were predicted on weekends (Table 2).

We also found two interesting patterns associated with the before-phase and after-phase of the events from our mixed-effects model. First, our top model showed a small but noticeable uptick in the mean number of checklists prior to the first eBird report of a rarity in the before-phase of the events (Fig. 2). We interpret this pattern as an artifact of some rarities being first reported independently of eBird checklists. That is, not all birders use eBird so some fraction of rarities are actually discovered prior to their first detection on an eBird checklist. Some rarities are first reported via other forms of communication within the birding community (e.g., word-of-mouth, email, social media, online birding forum, etc.). Nevertheless, our findings show that the number of checklists did not rapidly surge until the first detection of a rarity on an eBird checklist. This suggests that the draw of rarities is greatly influenced by their initial inclusion on eBird checklists, perhaps even more so than other forms of communication among birdwatchers. eBird rare bird alerts, which send hourly or daily electronic notifications of rarity reports within user-specified geographic areas (e.g., counties, states, etc.), are probably responsible.

Contrary to our hypothesis, we also found an unexpected drop in birder effort in the after-phase of events (Fig. 2). We predicted that the number of checklists would decay from a peak in effort back to the baseline number of checklists for a given location over time because of the decline in the number and interest of available rare bird chasers. However, our best model showed that the mean number of eBird checklists for a given location dropped below the baseline level of checklists submitted prior to the onset of events. We interpret this reduction in effort as a kind of cost-benefit analysis leading to ‘location fatigue’ where the absence of a desired rarity leads to a decision to spend birding-time elsewhere. In this way, the behavior is similar to how anglers may seek new fishing spots once a current spot has “run dry”. We noticed a marked reduction in the mean effort of eBirders across all events after the final detection of the rarity on a checklist. Because understanding the motives of participants has important ramifications for studies that incorporate community-contributed data (Bonney et al., 2009; Roy et al., 2012; Geoghegan et al., 2016), this pattern of location avoidance may be worth the attention of researchers and managers who rely on data collected via community-based science programs, such as eBird.

In our models, the average checklist rate for draw was driven by the additive influence of increasing year, distance to a small airport, and latitude (Tables 3 and 4). Draw was greater on average across our events as latitude increased. The clear geographical bias we found in our dataset may help explain this relationship, where mega-rarities tended to primarily cluster in extreme southern parts of the US. Indeed, 53% of the events occurred in extreme southern portions of Arizona and Texas, or in the state of Florida. Of the remaining 28 states where events occurred, no other individual states accounted for more than 5% of the events, with most states accounting for 1% of events or less—the notable exception being California, which accounted for 11% of the events in our dataset. Draw may therefore have been greater in higher latitude locations simply because rarities occur far less frequently in those states than in low latitude states, thus increasing their attractiveness to eBirders who chase rarities. This aggregation of rarities into a limited set of geographic locations is not a unique characteristic of the United States. Geographic boundaries, such as coastlines, peninsulas and offshore islands are famous for attracting birders seeking rare birds, especially during migratory seasons (Howell, Lewington & Russell, 2014). Whether the same patterns of birder behavior occur on other continents, however, remains unclear currently but should be quantifiable in the future as eBird is increasingly adopted as a global database for archiving bird observation data. The early adoption of eBird in the United States has established a sufficiently large repository of bird data spanning nearly two decades that allowed us to parameterize all the necessary components for measuring draw and decay rates. If other country-specific databases possess similar data requirements as eBird, our conceptual framework may be extended to other locations and birding cultures.

Our prediction that rarities discovered at sites nearer to large population centers would have larger draw was somewhat supported by the results. Draw was negatively influenced by distance to small airports, suggesting higher draw near discoveries in areas with some limited access to long-distance travel. The relationship with distance to airport suggests a link with travel and eBirder access is important because population size of nearest city was not included in our models as being influential to draw. Because many of the locations attracting the most rarities in our study area are often distant from the largest cities (e.g., southeastern Arizona, the Rio Grande Valley, and the Florida Keys), but are not remote, the influence of these travel variables may be specific to the United States. Evaluating the same variables in other countries where the distribution of rarities relative to human population centers differs could reveal other patterns of draw and decay. For example, Great Britain is famous for its attraction of rarities, while also being relatively small in size, which motivates a large proportion of its birding community to actively chase rarities (Sheard, 1999).

The year in which an event took place was an important predictor of draw as well as the number of checklists submitted before events. We interpret these significant positive correlations with year as indicating the consistent growth in the use and popularity of eBird with each passing year. The eBird database has dramatically increased in size over the 10-year period included in our study, from nearly 10 million bird observations submitted worldwide in 2008, to more than 100 million observations by the end of 2017 (Sullivan et al., 2009; eBird, 2017a; eBird, 2017b). It is thus unsurprising that more eBirders chased rarities over time given the increased number of eBird observations in general with each passing year. On average, only event duration was important for explaining rates of decay, which as expected became more gradual for longer events.

Our models indicated that year, Julian date, population of nearest city, distance to small airport, and a host of different land cover classifications were all important factors for explaining mean eBirder effort in the locations contained in our dataset prior to events (Table 3). While these factors may have been important in attracting or deterring eBirders to certain locations prior to events, most became inconsequential following rarity discoveries. This suggests that mega-rarities have a profound influence on the behavior of birders simply by virtue of being very rare, though the level of rarity (code-4 vs. code-5) did not influence the magnitude of draw. The inclusion of year as an important positive predictor of both the before-event effort and *draw* during events also underscores that while there may be more eBirders through time, mega-rarities have a strong influence on the behavior of birders.

Our findings did not indicate that species-specific covariates were influential factors in predicting the draw of rare birds, but some noteworthy cases are worth highlighting. Some events resulted in much greater draws. For instance, the 2014 documentation of a Whiskered Tern (*Chlidonias hybrid*) at Cape May Point, NJ resulted in a maximum daily value of 161 eBird checklists, compared to an average of 32 daily checklists per day prior to its discovery. Similarly, a Variegated Flycatcher (*Empidonomus varius*) at the South Padre Island Birding and Nature Center in coastal Texas, and a Eurasian Hobby (*Falco subbuteo*) in the Wa’atch River Valley of Washington’s Olympic Peninsula, resulted in respective maximum daily values of 103 and 101 additional checklists above mean daily rates prior to their discoveries. Thus, despite the overall lack of a strong signal for effects of species on draw and decay, some particular rarities have had especially strong effects on birding activity.

### Mythbusting the patagonia picnic table effect

The PPTE hypothesis dictates that increased effort and attention from rarity-chasers should equate to more rare bird discoveries than regular birding on average. In the United States, PPTE has gained widespread use and is hypothesized to happen regularly. However, our findings do not support the hypothesis. Although we found that the discovery of rarities does indeed change birder behavior (Fig. 2), we found little evidence for improvement in discovery rates of additional rare birds. Because each location has a different bird community, with different proportions of rare species, a fair comparison must control for such differences. Our comparison evaluated rarity discovery rates against each site’s own baseline level of birding activity. The baseline rate of rarity discovery at the geographic locations we studied was 8 per 1000 checklists submitted. The rate at which new rarities were found subsequent to initial rarity discovery was not significantly different from the baseline rate (Fig. 3). We conclude that despite the increase in birder effort and attention associated with chasing rarities, birders have no better chance to find a rarity when chasing a previously reported event-initiating rarity than to find one during routine (baseline) birding. Based on these findings, we conclude the PPTE hypothesis is not supported and thus not a common phenomenon.

Our results are based on data from events associated with the rarest of birds in the U.S. (ABA code-4 and -5 species). By not including events defined by the occurrence of code-3 species on eBird checklists, we acknowledge that our findings may be missing instances where increased effort from discoveries of code-3 species could be interpreted to have led to subsequent discoveries of other code-3 species, or code-4 and -5 species. We limited our investigation to events with the rarest species in the U.S., because some code-3 species, while considered rare, do indeed occur with some regularity in certain parts of the U.S., and may not evoke the same level of chasing behavior as species considered to be mega-rarities. Examples of such species include Aplomado Falcon (*Falco femoralis*), a Neotropical raptor that occurs with annual regularity in parts of coastal Texas, or Ruff (*Calidris pugnax*), an Afro-Eurasian shorebird that consistently occurs along both East and West US seaboards in small numbers each year. Even a code-3 species often attributed as important to the origination of the PPTE moniker, Black-capped Gnatcatcher (*Polioptila nigriceps*), consistently occurs annually in known locations in the mountains and canyons of southern Arizona. An investigation of how code-3 species discoveries lead to additional rarity discoveries would need to take many geographical biases into account and was beyond the scope of this study. Likewise, geographical locations outside the United States may have different frequency distributions of rarity occurrences, so the dynamics of PPTE-like scenarios may vary by geography.

## Conclusions

The growth in eBird over the last decade has demonstrated the platform’s ability to inspire an increasing number of observers who engage with and document avian biodiversity around the world. Indeed, we have learned that the draw of rare birds in the United States has increased with the rise in eBird use over the past decade, resulting in more eBirders chasing rarities soon after they are first detected and reducing attention to locations after rarity detections cease. As eBird continues to grow in use and popularity in the coming years more birders are likely to encounter and report rare birds, which undoubtedly will lead to an increasing number of eBirders who chase rarities, providing additional opportunities to evaluate factors that influence birder behavior and motivations. Our results do not support the supposition that rare bird discoveries regularly lead to increased discoveries of additional rarities, at least in our study area.

Further research that builds upon the findings we present in this paper is needed to understand the consequences of chasing behaviors, including the economic benefits from avitourism, potential impacts to species and habitats, and the carbon-footprint of birders who travel to chase rare birds. Other directions for future research would be to include rare bird events characterized by lower-threshold (code-3) species to understand geographic and species-specific variation in rarity discovery rates. Furthermore, reports of rarities may attract chasers with a wide range of experience levels. Because of the nature of the eBird checklist data we used, which did not contain metrics on observer experience-level, our analyses did not attempt to differentiate between the skill-level of individual birders. Investigations that take into account the observer experience-level or identification expertise may offer further insights into how these factors influence the detection of additional rarities. Additionally, investigations that expand the level of inference to include areas outside of the contiguous US would also allow for more generalizable findings for how rare bird reports influence behavior across a wider spectrum of birders around the world with differing social, economic, and cultural demographics.

##  Supplemental Information

10.7717/peerj.10713/supp-1 Figure S1 Examples of non-convergent rare bird events that could not be fit using exponential decay models

10.7717/peerj.10713/supp-2 Table S1 Supplemental Tables

## Additional Information and Declarations

### Data Availability

The following information was supplied regarding data availability: The raw data and models are available in the Supplemental Files.
