# Supplementary figures and images for: The influence of rare birds on observer effort and subsequent rarity discovery in the American birdwatching community
Source: PeerJ. 2021 Jan 21;9:e10713. doi: 10.7717/peerj.10713 (PMC7827972; doi:10.7717/peerj.10713)

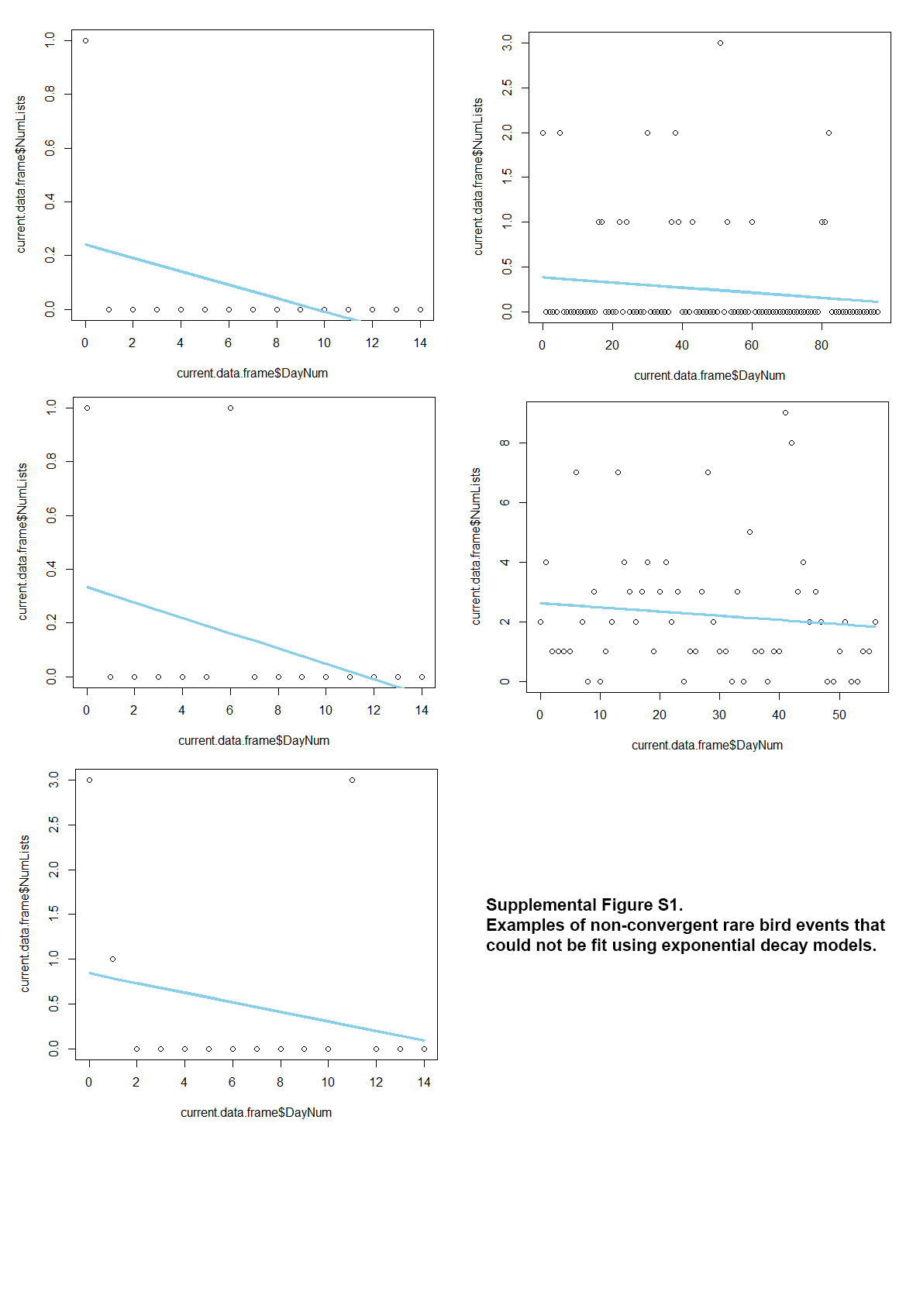

Supplement: Figure S1 [file peerj-09-10713-s001.png]
